# Binaural-cue reweighting induced by discrimination training

**DOI:** 10.3758/s13414-025-03082-x

**Published:** 2025-05-14

**Authors:** Maike Klingel, Udbhav Singhal, Aaron R. Seitz, Norbert Kopčo

**Affiliations:** 1 Acoustics Research Institute, Austrian Academy of Sciences, Vienna, Austria; 2 Pavol Jozef Šafárik University, Košice, Slovakia; 3 Northeastern University, Boston, MA USA

**Keywords:** Spatial hearing, Binaural-cue reweighting, Interaural time difference, Interaural level difference, Trading ratio, Signal detection theory modelling

## Abstract

When localizing sounds, listeners combine the two binaural cues interaural time and level difference (ITD and ILD). The relative weight assigned to each cue is frequency dependent, with ITDs dominating at low and ILDs at high frequencies. However, this weighting changes (e.g., depending on room reverberation or cue reliability). To achieve better spatial hearing in various listener populations, changing the weighting might be advantageous. Previous studies showed that such changes can be induced (e.g., using a lateralization training with visual reinforcement in virtual reality). Here, a new training procedure is introduced, based on a simple auditory-only discrimination task. An experiment evaluated the procedure, consisting of a pretest, three training sessions, and a posttest. Subjects were divided into three groups—one trained by reinforcing the ILDs, one by reinforcing the ITDs, and one no-training control. The training consisted of an adaptive staircase of relative discrimination trials. Stimuli were two consecutive narrow-band noise bursts (2–4 kHz), each presented with a different combination of ITD and ILD. Participants’ task was to indicate the perceived location of the second noise burst versus the first. During training, feedback was provided requiring the subject to imagine the sound moving in the trained cue’s direction. We observed an increase in reinforced-cue weight for both training groups, but not in the control group, that continued during all three training sessions. Thus, this training method is effective for reweighting in both directions. Moreover, it is individualized, and, since it does not rely on sophisticated equipment, it can be easily accessible for a range of listeners.

## Introduction

Spatial hearing is an important part of our everyday life, as it enables us to localize sound sources and improves speech understanding in complex environments (Litovsky et al., 2021). This study focuses on spatial hearing in the horizontal plane. Normal-hearing listeners rely on two binaural cues for horizontal localization—namely, the interaural time difference (ITD), the difference in the arrival time of the sound to each ear, and the interaural level difference (ILD), the difference in sound pressure level received at the two ears (e.g., Stecker & Gallun, 2012). We are interested in how the contribution of these two cues to localization can be modified by training. Such a modification might benefit sound localization in challenging environments in which the contribution of the binaural cues is often not weighted optimally (e.g., Ihlefeld & Shinn-Cunningham, 2011) or with hearing devices such as cochlear implants which limit access to one of the cues (e.g., Laback et al., 2004).

While ITDs dominate the percept at low frequencies (and broadband sounds), ILDs dominate for high frequencies. This is known as the duplex theory of sound localization (Ahrens et al., 2020; Klingel & Laback, 2022; Macpherson & Middlebrooks, 2002; Strutt, 1907). Traditionally, this weighting of the binaural cues has been measured by letting participants adjust one of the cues until a stimulus with the other cue fixed at a certain magnitude is perceived centrally, yielding the trading ratio (e.g., Deatherage & Hirsh, 1959). However, this method leads to a stronger weighting of the to-be-adjusted cue, either due to shifted attention (Lang & Buchner, 2008) or cue-specific adaptation (Moore et al., 2020). Alternatively, binaural-cue weights have been measured by asking participants to lateralize auditory stimuli containing binaural cues that correspond to different spatial locations. The weighting can then be inferred by comparing the response location to the locations corresponding to each of the cues (e.g., Klingel et al., 2021; Macpherson & Middlebrooks, 2002). This approach, however, requires sophisticated equipment to accurately record response locations, such as virtual reality equipment.

While the binaural-cue weighting mainly depends on the sound’s frequency content, there are other influencing factors as well. For instance, the weighting also depends on the overall level of the sound (David et al., 1959; Deatherage & Hirsh, 1959), the interclick interval of click trains (Stecker, 2010), or room acoustics (Ihlefeld & Shinn-Cunningham, 2011; Rakerd & Hartmann, 2010). Additionally, substantial variation is observed across participants (Klingel et al., 2021; Macpherson & Middlebrooks, 2002).

This dependence on stimulus, environmental, and personal factors is not surprising, given that listeners adapt to cue alterations when localizing sounds (see Carlile, 2014; King et al., 2011; Wright & Zhang, 2006, for reviews). Such adaptation can either be a result of remapping (i.e., building new associations between sound localization cues and their corresponding locations in space; e.g., Shinn-Cunningham et al., 1998) or reweighting (i.e., increasing the relative weighting of unaltered or reliable cues compared to altered or unreliable cues). Several studies, for example, report a stronger weighting of monaural, spectral-shape cues (i.e., the directional filtering of the pinnae) compared with binaural localization cues for horizontal sound localization after wearing unilateral earplugs (Keating et al., 2013; Kumpik et al., 2010; van Wanrooij & van Opstal, 2007). This is interesting given that monaural cues are mainly used for vertical-plane localization and usually do not contribute to horizontal-plane localization except for resolving front/back confusions, when binaural cues are available (Macpherson & Middlebrooks, 2002; Slattery & Middlebrooks, 1994). Furthermore, participants were shown to reweight the two binaural cues ITD and ILD after one of the cues was reinforced during a lateralization (i.e., in-head localization in the horizontal plane; Plenge, 1974) training in a virtual audio-visual environment (Klingel et al., 2021), although this depended on the auditory stimulus used (Klingel & Laback, 2022). Additionally, binaural-cue reweighting might depend on the employed task. Kumpik et al. (2019) observed an increase in ILD weighting for a condition with stable ILDs and randomized ITDs, but no increase in ITD weighting for the opposite condition (i.e., stable ITDs and randomized ILDs), after participants completed a visual oddball task (i.e., the auditory stimuli were task-irrelevant). Furthermore, Jeffress and McFadden (1971) did not observe any change in binaural-cue weights after participants completed a left/right discrimination training in which the ITD and ILD of a noise band centered at 500 Hz favored opposite ears. However, given that the auditory stimuli used in the latter two studies included frequency regions where temporal fine-structure information is available, which, according to Klingel and Laback (2022), prevents binaural-cue reweighting, it is unclear whether a lack of reweighting resulted from the employed task or from the auditory stimuli used.

Here, we introduce a training protocol for binaural-cue reweighting that uses a simple left/right discrimination task and concurrent 2-down-1-up adaptive staircases to induce an increase in the ITD or ILD weight. We evaluate it on auditory stimuli for which binaural-cue reweighting has been successfully induced using an audio-visual lateralization training (Klingel et al., 2021) to test whether reweighting can be obtained using this protocol that does not require sophisticated virtual reality equipment (and thus can be implemented on a regular desktop computer/tablet/cell phone). The protocol uses a two-interval, relative discrimination task instead of a one-interval task (as in Jeffress & McFadden, 1971), which allows us to train many configurations across the whole spatial range, since we are not restricted to azimuths close to the midline. It also includes corrective measures (i.e., repeating the auditory stimulus with the “correct” response shown on screen) in addition to feedback after each response in case participants responded “incorrectly,” to ensure listeners’ attentiveness to the task. Finally, we introduce a signal detection theory–based model (Durlach & Braida, 1969) to describe the reweighting data, providing a more generalizable and robust estimate of the relative weighting of the ITD/ILD cues.

## Methods

### Participants

In total, 36 participants (age range: 19–58 years; 18 women) completed the experiment. Fourteen participants were assigned to the ITD group and 11 to the ILD group (each group trained to increase the respective cue weight), while 11 participants were in a no-training control group. All but three participants had audiometrically normal hearing (≤ 20 dB HL threshold at frequencies between 250 and 8000 Hz). The remaining three participants had thresholds ≤ 35 dB HL, but thresholds ≤ 20 dB HL at the center frequency of the stimuli used in this study. All subjects gave informed consent, and the experiment was approved by the ethical committee of UPJŠ.

### Apparatus and stimuli

During the experiment, participants were seated at a desk inside a sound booth containing a display, keyboard, and headphones. The experiment was controlled by a PC placed outside the booth and running a custom-written software in MATLAB with Psychtoolbox-3 to control the experiment, generate stimuli, and collect responses. Binaural auditory stimuli were generated using an external sound card (RME Fireface 400) and presented via headphones (Sennheiser HD 800 S was used for the control and ILD groups and Audeze LCD-X was used for the ITD group, which performed the study after the other groups).

**Fig. 1:**
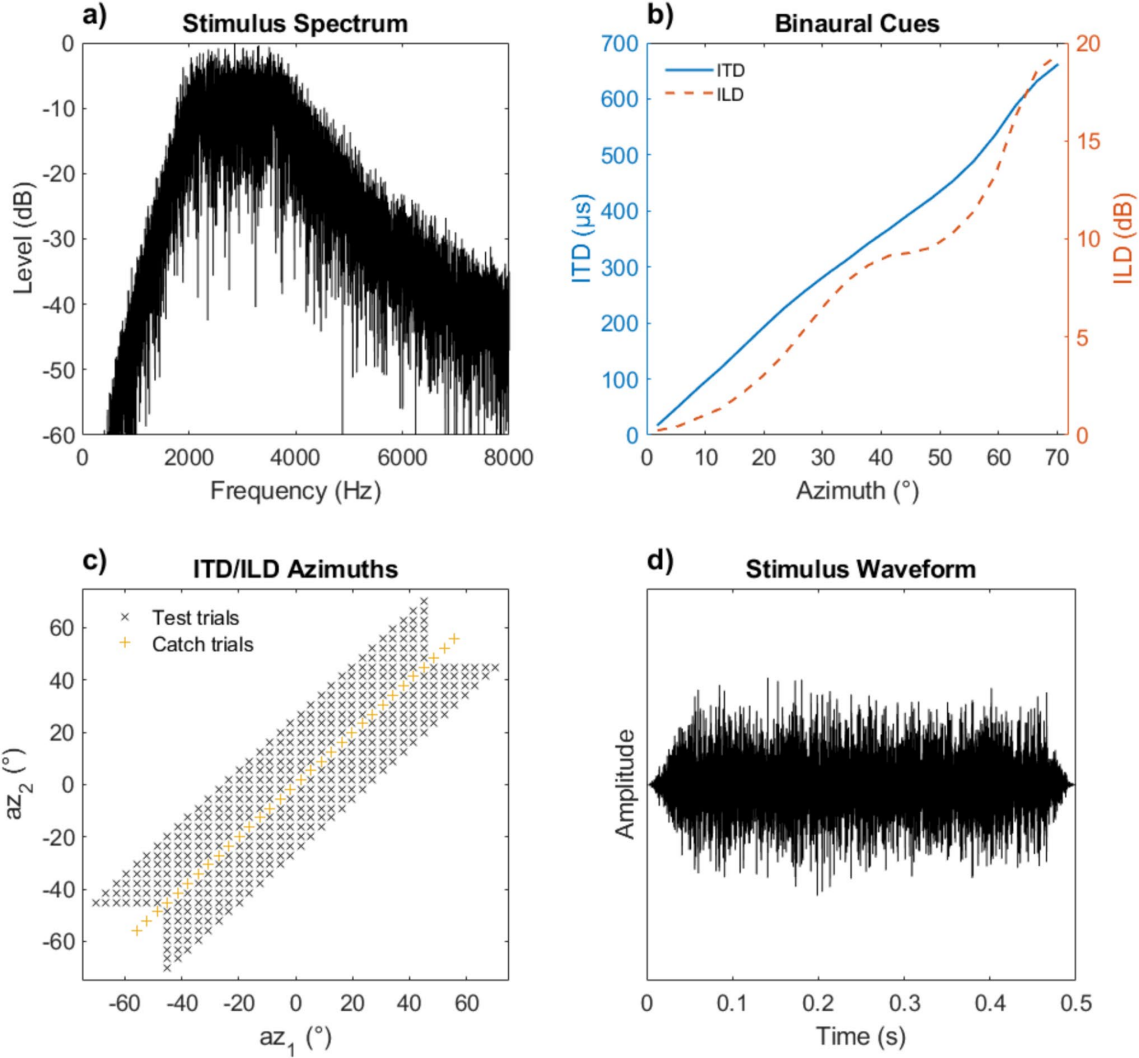
Auditory stimuli. Panel **a)** shows the stimulus spectrum, panel **d)** shows the stimulus waveform, panel **b)** shows the relationship between the binaural cues and their corresponding azimuth, and panel **c)** shows the combinations of azimuths (and hence combinations of ITD and ILD) used during the pre- and posttest. The black “x” symbols in panel c) show the inconsistent azimuth combinations used in test trials to determine the binaural-cue weighting. Az_1_ was always assigned to ITD, and az_2_ to ILD (or vice versa). The yellow “ + ” symbols show the consistent combinations used in the catch trials to determine if participants performed the task as intended. During training, a subset of these combinations was used in each run, determined by the adaptive procedure. (Color figure online)

Each auditory stimulus consisted of two 500-ms white noise bursts, including 50-ms on/off ramps (Fig. 1d), as used in Klingel et al. (2021), with an interstimulus interval of 0 ms. The bursts were randomly generated for each trial and filtered by a 2–4 kHz Butterworth band-pass filter (F_C_ = 2.8 kHz; roll-off 30 dB/oct; Fig. 1a and d). Additionally, interaural time differences (ITDs) ranging from − 662 to + 662 µs and interaural level differences (ILDs) ranging from − 19.4 to + 19.4 dB were imposed on the filtered noise bursts. These ITD and ILD cues corresponded to an azimuthal range spanning from − 70.2° to + 70.2° as estimated by Xie (2013; Fig. 1b).^1^ Possible combinations of azimuths simulated by ITD and ILD during testing and training are shown in Fig. 1c. To discourage listeners from using absolute levels for determining the stimulus azimuth, the presentation level of each noise burst was independently roved (rove level uniformly distributed between ± 2.5 dB).

### Procedure

The experiment was conducted on 3 consecutive days. On the first day, all participants underwent the pretraining to get familiar with the task and completed the first assessment (pretest). Furthermore, participants belonging to one of the training groups had their first training session. On the second day, only participants belonging to one of the training groups attended and completed the second training session. On the third day, participants belonging to one of the training groups had their third training session, and all participants underwent the second assessment (posttest).

#### Pretraining

**Fig. 2:**
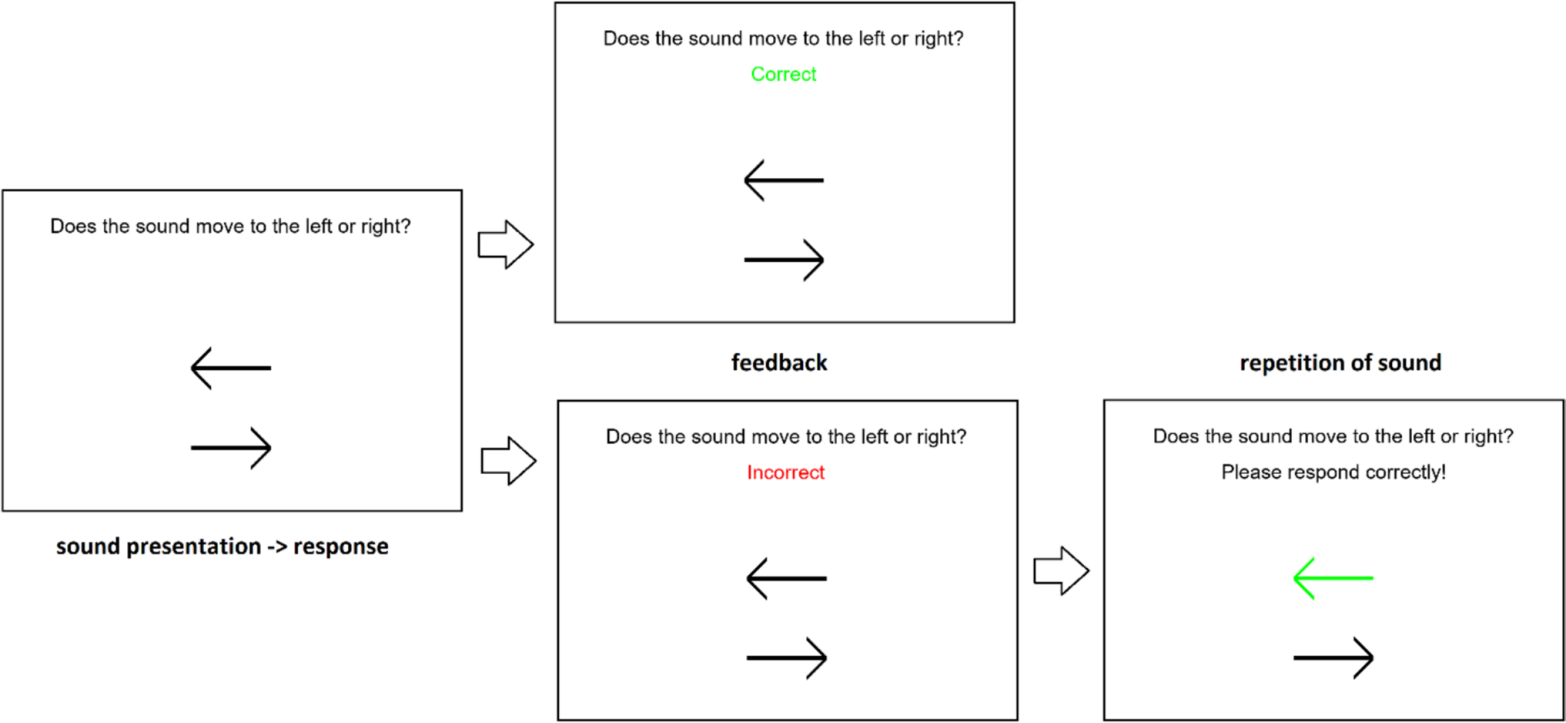
On-screen prompts during training and pretraining trials (during test trials, only the first screen appeared). (Color figure online)

The procedure of the pretraining consisted of 50 trials using consistent-cue stimuli (each stimulus consisted of two consecutive noise bursts, each containing an ITD and ILD corresponding to the same azimuth). The first noise burst had a randomly chosen azimuth between ± 45° (with 3.6° spacing) and the second noise burst was then shifted to the left or right by 10.8°. Participants had to indicate whether the sound moved to the left or right by pressing the respective arrow key. They received feedback (correct/incorrect) after each response. If they responded incorrectly, the auditory stimulus was presented again with the correct response shown on screen and participants had to respond correctly in order to move on to the next trial (see Fig. 2). If the mean accuracy across the 50-trial run was below 75%, the pretraining run was repeated until the threshold was reached. Two participants of the ILD group, two participants of the ITD group, and none of the control group had to repeat the pretraining.

#### Testing

**Fig. 3:**
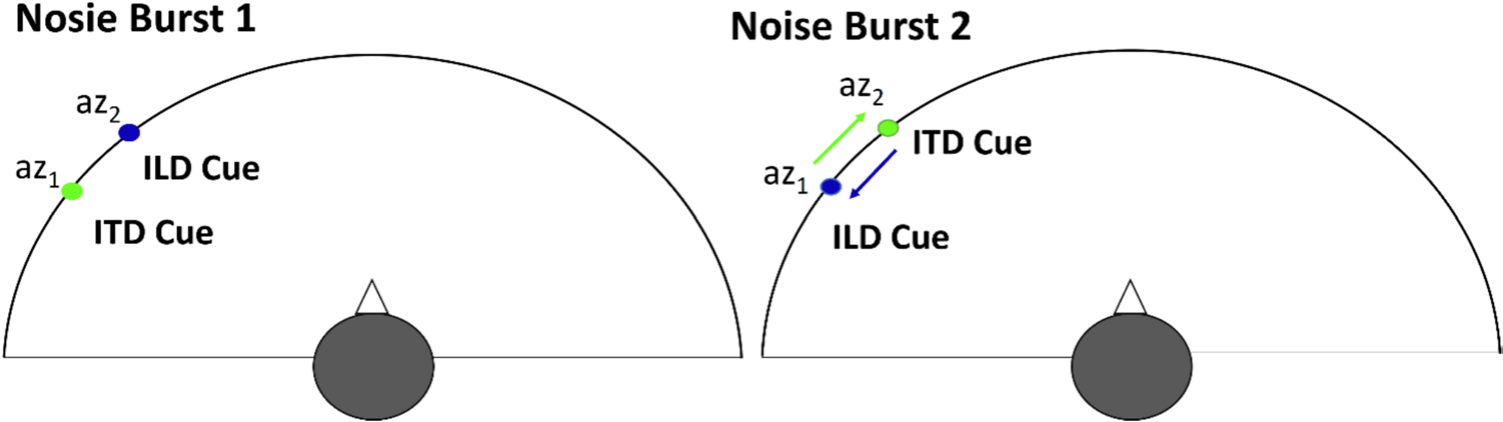
Design of the stimulus in a pre-/posttest trial. Each stimulus consisted of two consecutive noise bursts, one containing ITD corresponding to az_1_ and ILD to az_2_ (or vice versa) and the other one with the cue azimuths reversed. (Color figure online)

During the test runs, each trial consisted of the presentation of a stimulus (i.e., two consecutive noise bursts) followed by a response in which the listener indicated whether the second burst was perceived to be left or right of the first (i.e., whether the sound moved to the left or right). Participants did not receive feedback (i.e., only the first screen from Fig. 2 was shown). The same inconsistent ITD/ILD combinations as in Klingel et al. (2021) were used (black “x” in Fig. 1c). Each stimulus used two azimuths, az_1_ and az_2_ (Fig. 3). One of the azimuths (az_1_ or az_2_) was selected pseudo-randomly on each trial from the range ± 45° (with step of 3.6°) and the other azimuth (az_2_ or az_1_) was shifted to the left or right of the first one by between 3.6° and 25.2°, again with a uniform 3.6° spacing (the difference between the azimuths is referred to as the cue disparity).

For the first noise burst, the ITD corresponded to az_1_ and the ILD corresponded to az_2,_ while for the second noise burst the azimuths were swapped such that the ILD corresponded to az_1_ and the ITD corresponded to az_2_ (or vice versa). Since we switched both azimuths, the cues were shifted in opposite directions by the same azimuth. It was assumed that the perceived direction of motion is indicative of which cue contributed more to the azimuthal percept. That is, if the ILD is weighted more, the participant should hear the sound as moving in the direction the ILD is moving (and vice versa if ITD is weighted more).

There is no objectively correct response in this task since it depends on the binaural-cue weighting. So, we additionally included catch trials with consistent-cue combinations to monitor whether participants performed the task correctly. In the catch trials, the first noise burst corresponded to an azimuth between ± 45° (uniformly distributed, 3.6° spacing) and the second noise burst corresponded to an azimuth shifted by 10.8° either to the left or right regarding the first burst. That is in the catch trails, both ITD and ILD moved either to the left or to the right.

Each testing (pre-/posttest) session consisted of a total 892 trials (including 52 catch trials) with four repetition sets of all 210 possible az_1_/az_2_ combinations (assuming az_2_ > az_1_) as the assignment of ITD vs ILD to az_1_ vs. az_2_ of the first stimulus was randomized on each trial (Fig. 1c, all black “x” symbols above the diagonal).

#### Training

**Fig. 4:**
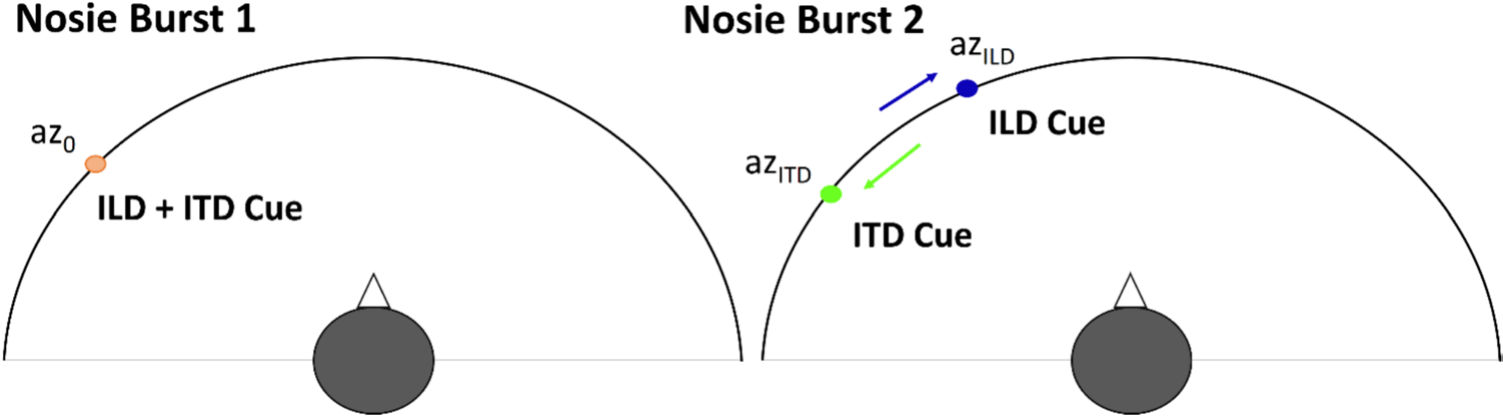
Design of the stimulus in a training trial. Each stimulus consisted of two consecutive noise bursts. The first burst had the ITD and ILD corresponding to the same azimuth az_0_. The second burst had the ITD and ILD shifted by variable amounts (see text for details). (Color figure online)

Each training session consisted of three interleaved adaptive staircases (one each for cue disparities of 18°, 21.6°, and 25.2°), in which the trained cue (e.g., ITD for the ITD group) values were set adaptively, while the nontrained cue value was determined by the disparity. The stimulus of each training trial again consisted of two noise bursts (Fig. 4). For the first burst, azimuth az_0_ between ± 30.6° with a 3.6° spacing was chosen randomly and both the ITD and ILD corresponded to that azimuth (i.e., a consistent-cue combination was presented; yellow “ + ” in Fig. 1c). The second burst had an inconsistent-cue combination. The trained cue (either az_ITD_ or az_ILD_, depending on the group) was shifted to the left or right (chosen randomly) from az_0_ by an amount (i.e., offset) that was manipulated adaptively using a 2-down-1-up procedure, starting at 32.4° and varying in the range of 3.6° to 32.4° in steps of 3.6°. The untrained cue was always shifted in the opposite direction to the trained cue such that the offset of az_ILD_ from az_ITD_ (i.e., the cue disparity) was constant (18°, 21.6°, or 25.2°) for each adaptive staircase track. Note that at the beginning of each track both ITD and ILD actually moved in the same direction, as the trained cue offset of 32.4° was larger than the disparity, ensuring that the task could be initially solved irrespective of the binaural-cue weighting. Which of the three interleaved staircases was advanced on a given trial was chosen randomly.

After the presentation of each stimulus, the participant again responded by indicating the perceived shift direction (left or right), followed by feedback (Fig. 2). If the response matched the trained cue direction, the participant received the feedback “correct.” If it did not, the participant received the feedback “incorrect,” and the stimulus was played again with the “correct” response shown on screen. The participant was asked to imagine the perceived sound moving in the “correct” direction and respond accordingly. Then, the next trial was initiated.

Each training session consisted of 500 trials combined across the three adaptive tracks and took approximately 30–40 min to complete.

### Analysis

The following analyses were performed for the testing data. For the catch trials, the proportion of correct responses was calculated for all three groups. For the inconsistent-stimulus test trials, the proportion of trials in which participants’ responses followed the ILD azimuth (P_ILD_) was computed for all stimulus azimuths and cue disparities (note that P_ITD_ = 1 – P_ILD_). P_ILD_ is an estimate of the ILD/ITD weight such that the value of 0.5 means equal weighting, and it was evaluated separately for different az_1/2_ combinations.

The P_ILD_ is a straightforward estimate of the binaural-cue weight from the current discrimination data. However, it has several disadvantages (e.g., it can vary depending on cue disparity; it tends to be closer to 1 or 0 at large disparities and closer to 0.5 at smaller disparities, independent of the actual relative ILD/ITD weight). Also, it is noisier for smaller cue disparities as those responses are more likely to be dominated by the noise in the internal representation of the stimulus. Therefore, a model based on the 2I-2 AFC signal detection theory model (Durlach & Braida, 1969) was derived that provides a single ILD/ITD weight measure, similar to the standard trading ratio (Stecker, 2010), for all combinations of azimuths and disparities. Using the model modifications and assumptions defined in Kopčo et al. (2012), the following equation defines the percentage of responses following the ILD, P_ILD_, as a function of the relative weight *w*_*LT*_:

1 $${P}_{ILD}=\frac{1}{\surd 2\pi } {\int }_{-\infty }^\frac{d}{2}{e}^{\frac{{-t}^{2}}{2}} dt, \text{where }d={w}_{LT}\left|{az}_{2}-{az}_{1}\right|$$

Here, *d* is a *d’*-like measure that represents the sensitivity to ILD versus ITD (however, it can be both positive, when the responses follow ILD, and negative, when the responses follow ITD). It is assumed to be proportional to *w*_*LT*_ scaled by the disparity between the two stimuli. Thus, w_LT_ expresses the relative ILD/ITD weight for azimuthal disparity of 1° and is in units of deg^−1^. The value of w_LT_ is 0 when the cues are weighted equally, positive when ILD is weighted more and negative when ITD is more. The model’s w_LT_ was fitted on the P_ILD_ data averaged across azimuths since the difference between pre- and posttest values of P_ILD_’s is approximately independent of azimuth. We used nonlinear fitting, optimizing the weighted root-mean-square error (RMSE) between the predicted versus measured P_ILD_ to obtain the fits that mostly rely on the larger disparities, given that the small-disparity P_ILD_’s are noisier.

For the training data, we analyzed the trained-cue offset (i.e., the difference between the trained-cue azimuth of the second noise burst and the azimuth of the first, consistent-cue noise burst of each stimulus) at the staircase reversals (after skipping the first 20 trials, which, on average, included two reversals, where the data can be particularly noisy). We averaged the trained-cue offset in 10-reversal bins. Four such bins were considered for each adaptive track, session and group (note that the actual number of reversals varied across the tracks, but each of them had sufficient number of reversals to create four bins).

Unless specified otherwise, repeated-measures or mixed analyses of variance (ANOVAs) were used for statistical significance testing, as implemented in CLEAVE software (Herron, 2005).

## Results

### Catch trials

To assure that the relative weight values are not affected by fluctuation in subjects’ attention or overall performance from pretest to posttest, we first analyzed the catch trial direction discrimination performance. Discrimination accuracy was similar across the three subject groups, and both tests (across-subject average percentage correct in pretest vs. posttest was 71.5% vs. 74.5% in the control group, 80% vs. 79.5% in the ILD group, and 77% vs. 78% in the ITD group). Confirming this, a 3 (group) × 2 (time) mixed-design ANOVA found no significant differences (all *p*-values larger than .360).

### Testing data

**Fig. 5:**
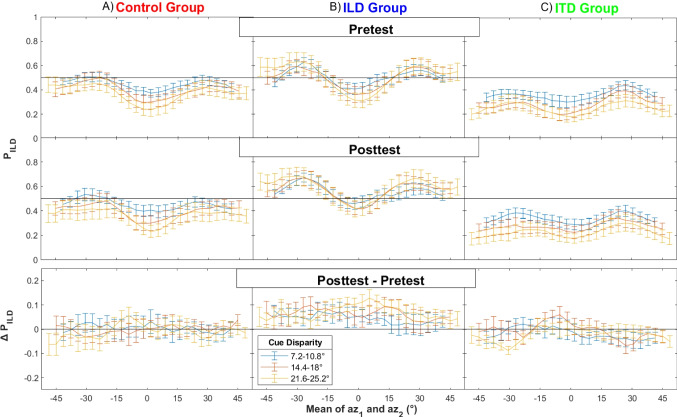
Proportion of responses that followed the ILD, P_ILD_, as a function of azimuth (mean of az_1_ and az_2_) plotted separately for the three groups (columns), and two test sessions and their difference (rows). Line color represents cue disparities grouped into small (blue), medium (red), and large (yellow). (Color figure online)

The effectiveness of the discrimination training was first analyzed by evaluating the P_ILD_ measure separately for all combinations of azimuths az_1_ and az_2_, averaged across the trials differing only in the order of assignment of ITD/ILD to az_1/2_. Figure 5 plots P_ILD_ as a function of the average az_1/2_ azimuth, separately for the small (7.2–10.8°), medium (14.4–18°), and large (21.6–25.2°) cue disparities, represented by line color. Each column of panels represents a different group, while the rows represent the pretest and posttest session, as well as the post versus pre comparison.

The pre- and posttest results show an overall preference for ITDs (i.e., P_ILD_s smaller than 0.5 in upper and middle row panels) except in the ILD group, for which the values fluctuate around 0.5 (upper and middle panel in Column B). Furthermore, ILDs appear to be weighted more for lateral compared with central azimuths (i.e., P_ILD_s are larger at azimuths around ±30°). This pattern is more pronounced for larger cue disparities (i.e., yellow lines are further away from 0.5 than blue lines). When comparing the pre- versus the posttest (∆P_ILD_ in the bottom row), there was no systematic difference for the control group (i.e., values fluctuate around 0 in the bottom panel of Column A). Successful training for the ILD group would be shown by positive ∆P_ILD_s, and for the ITD group by negative ∆P_ILD_s. For the ILD group, ILDs were indeed favored more often in the post- compared with the pretest, at all disparities (Column B). For the ITD group (Column C), ILDs tended to be favored less often in the post- compared with the pretest for large cue disparities, but the pattern appears to be weaker and less clear than in the ILD group.

### Signal detection theory model

**Fig. 6:**
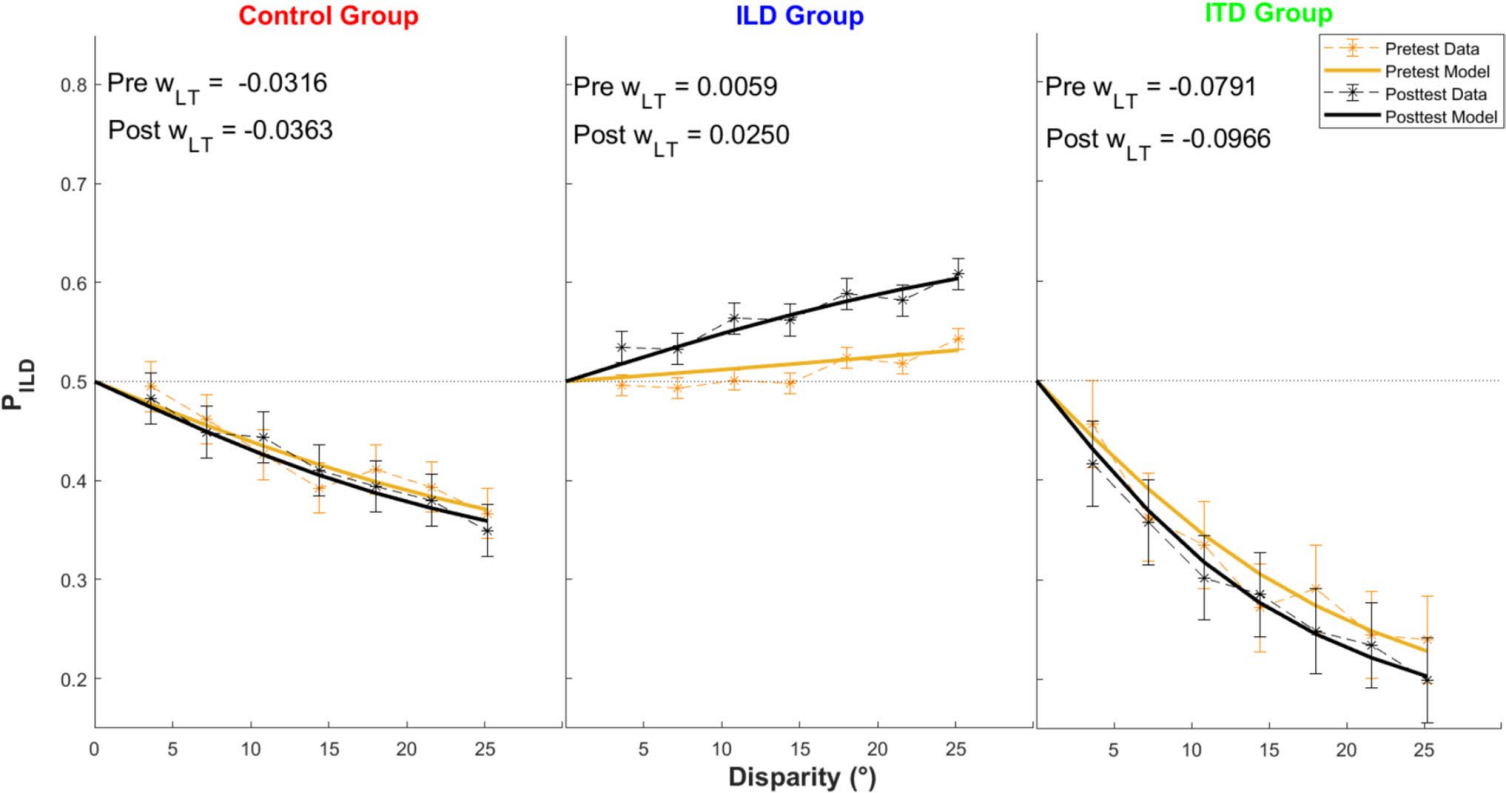
Across-subject average P_ILD_s and model fits as a function of cue disparity, averaged across azimuths. Error bars show the standard error of the mean. Average fitted w_LT_ values are shown in the insets. (Color figure online)

Since P_ILD_ values and their reliability vary with cue disparity, the primary evaluation of the effectiveness of the training was performed on the binaural-cue weight estimates, w_LT_, obtained by fitting a signal detection theory–based model to the data. To validate the fits, Fig. 6 visualizes the model fit for the three groups. It plots the across-subject average P_ILD_ as a function of cue disparity (collapsed across azimuths; dashed lines) along with the across-subject average of the model fits to each individual (solid lines). The model fits are very accurate (across-subject average coefficient of determination of the individual fits, *r*^*2*^, is .379).

**Fig. 7:**
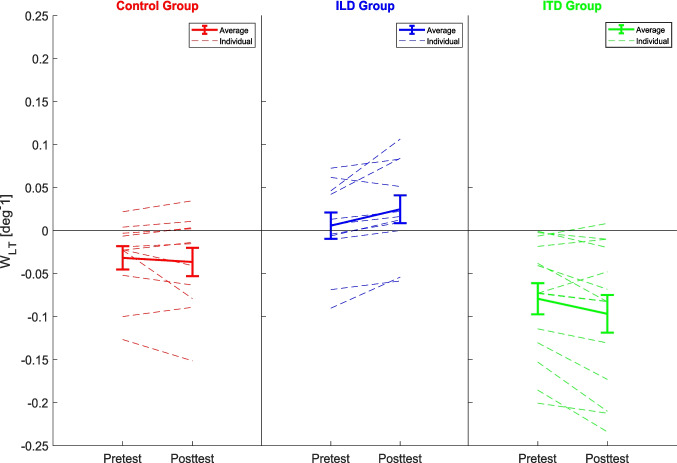
Pretest and posttest binaural-cue weights (w_LT_) estimated for individual participants and averaged within groups. Error bars show the standard error of the mean. (Color figure online)

Figure 7 plots pre- and posttest w_LT_s obtained by the models for each group. Dashed lines show the results for individual participants and solid lines represent group averages (the average values are also stated in the insets of Fig. 6). A 3 (group) × 2 (time) mixed-design ANOVA showed a significant interaction, *F*(2,33) = 8.54, *p* = 0.001, η_p_^2^ = 0.011, and a significant main effect of group, *F*(2,33) = 8.96, *p* < 0.001, η_p_^2^ = 0.341. Follow-up pairwise comparisons showed that the effect of training was significantly different between all three group pairs (control vs. ILD, control vs. ITD, and ILD vs. ITD group) with Bonferroni corrected *p* ≤ 0.002. The average difference in weights were − 0.005 deg^−1^ for the control group, 0.020 deg^−1^ for the ILD group and − 0.018 deg^−1^ for the ITD group, suggesting that the training was approximately equally efficient (with an opposite sign) in the two training groups. The main effect of group is in part driven by the training effect (ILD group was shifted up in the posttest, while the ITD group was shifted down), and in part by the random assignment of subjects into the groups (even in the pretest, the ILD group is on average more positive than the ITD group, with the control group falling in the middle). While this group difference is unexpected, it is not likely to drive the differential learning effect across the groups as the effect is present in most subjects in both training groups and not concentrated on the outliers (i.e., the ILD subjects with the highest pretest w_LT_ or the ITD subjects with the lowest w_LT_).

### Training data

**Fig. 8:**
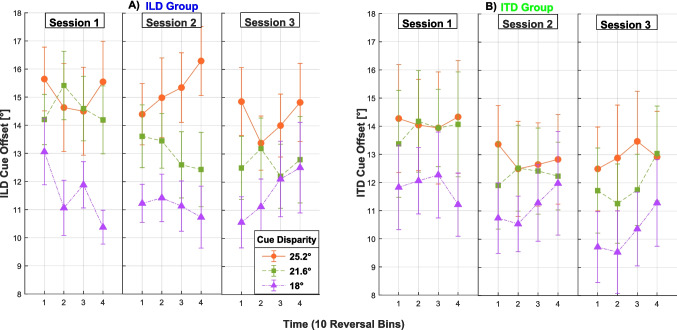
Trained-cue offsets in 10-reversal bins during the adaptive training runs, plotted separately for each session (column) and cue-disparity adaptive track (color). (Color figure online)

To examine how the training progressed within and across training sessions, we analyzed the trained-cue offset (i.e., the difference between the trained-cue azimuth of the second noise burst and the azimuth of the first, consistent-cue noise burst of each stimulus) at the adaptive track reversals. Figure 8 shows the average trained-cue offset in 10-reversal bins for the first four bins of each adaptive run of each training session, separately for each trained cue disparity/adaptive track (shown with differently color-coded lines). Smaller offsets indicate better performance. As expected, the offset is larger for larger cue disparities (e.g., for orange vs. purple lines), since the untrained cue “pulls” the percept in the other direction by the largest amount. No systematic pattern was observed across bins within sessions, but there was an improvement across sessions. Confirming these observations, a 2 (group) × 3 (session) × 3 (cue disparity) mixed-design ANOVA yielded significant main effects of session, *F*(2,46) = 8.50, *p* =.001, η_p_^2^ =.012, as well as cue disparity, *F*(2,46) = 44.70, *p* <.001, η_p_^2^=.051. There was no significant effect of group, suggesting a similar learning trajectory in the ILD and ITD group. The offset (averaged across bins, disparities and groups) was 13.50° in Session 1, 12.55° in Session 2, and 12.20° in Session 3. Thus, there appears to be a trend that the improvement across session was larger between Sessions 1 and 2 (0.95°) than between Sessions 2 and 3 (0.35°). However, Bonferroni-corrected pairwise comparisons between the three sessions did not find any significant differences, indicating that that trend is not significant. Instead, the fact that the improvement was present even between Sessions 2 and 3 indicates that the overall training effect might have been even larger if the training continued for more sessions.

## Discussion

We tested and evaluated a simple left/right discrimination training to induce binaural-cue reweighting as well as a measurement tool for binaural-cue weights that can be run on a regular desktop computer or even a tablet or a cell phone.

### Binaural-cue reweighting from pretest to posttest

It has previously been shown that the weighting with which the binaural cues ITD and ILD are combined to form an azimuthal percept can be changed using a lateralization training in a virtual audio-visual environment, if the auditory stimuli meet certain criteria (i.e., sufficiently high frequencies to increase the ILD weighting and sufficiently low frequencies without including frequency regions providing fine-structure ITD cues to increase the ITD weighting; Klingel & Laback, 2022; Klingel et al., 2021). These criteria suggest that reweighting relies on envelope-ITD cues. The discrimination training introduced here has several advantages compared with the Klingel et al., (2021, 2022) visual-feedback lateralization training. First, since it is adaptive it provides individualized training independent of the initial weight for any individual. Also, it is more robust in that it does not depend on the accuracy of individualized spatial simulation. Specifically, when nonindividualized HRTFs are used to derive binaural cues corresponding to a specific azimuth (and these values are then simply imposed on the stimulus without HRTF filtering, as was the case both in the previous and the current study), then the correspondence might not be correct for all individuals. And, the visual feedback used for training might not actually align with the trained cue, making the training less effective. On the other hand, the discrimination training used here only depends on relative differences in the cue values between the two noise bursts, which are always correct even if the absolute values do not point to the correct azimuth for a given individual. Overall, though, since the two studies used different performance measures, it is not possible to directly compare the induced strength of reweighting, to answer the key question of which of the training protocols is more effective. The signal-detection-theory model introduced here to estimate the relative weight provides a first step towards converting the different weight measures to a comparable estimate (e.g., the standard “trading ratio”; Stecker, 2010), which would allow us to evaluate the effectiveness also of other training protocols (e.g., Kumpik et al., 2019).

Studies using other tasks to induce binaural-cue reweighting failed to produce consistent results (Jeffress & McFadden, 1971; Kumpik et al., 2019). However, since their auditory stimuli did not meet the abovementioned criteria, it is unclear whether the observed lack of reweighting (or increased ILD weighting for both the ILD and the control condition) was due to the task or stimuli used. The present study addressed this question by using auditory stimuli for which binaural-cue reweighting has previously been induced successfully. The results suggest that both the ITD and the ILD weighting can indeed be increased for 2–4 kHz noise using a simple left/right discrimination training. In addition to the frequency region of the auditory stimuli, our training task differed from Jeffress and McFadden’s (1971) discrimination training in some aspects that may have further facilitated reweighting: We used a variety of spatial configurations instead of stimuli close to the midline only (and therefore close to the binaural-cue threshold, which may not have been salient enough) and provided multi-modal feedback while requiring a corrective response after “incorrect” responses.

Kumpik et al. (2019), who observed an increase in ILD weights in their randomized-ITDs condition but no change in ITD weights in their randomized-ILDs condition, also observed an increase in ILD weights for a control condition, making it difficult to attribute the observed binaural-cue-weight change to the training manipulation. Our no-training control group, on the other hand, did not show a change in binaural-cue weights, suggesting that the presently observed effects in the ITD and ILD groups are induced by the training itself. It should be noted that the three groups showed slightly different pretest performance, with the ITD group below and ILD group above the control group. This was unexpected given that all three groups completed the exact same experimental protocol up until the training, except that the data of the ITD group was collected at a later time point than the other two groups and with different headphones, which is unlikely to cause the differences. Importantly, this should not have facilitated the increase in ITD weights from pre- to posttest observed in the ITD group or the increase in ILD weights in the ILD group, as lower pretest w_LT_s instead leave less room for a further decrease in the weight (as desired for the ITD group), and similarly higher pretest w_LT_s leave less room for a further increase in the w_LT_ of the ILD group.

### Improvement across training sessions

While Klingel et al. (2021) only observed reweighting from the pretest to the first training session and no further improvement across sessions, the present study shows improvement across all three training sessions. The lack of improvement within session suggests that reweighting required consolidation overnight. Since a plateau was not yet reached during the three training sessions, further discrimination training might have continued to show effects, even though this was not the case for the lateralization training. In addition to the difference in responses (lateralization vs. discrimination), the training tasks of the two studies differed in the training mode: The lateralization study used a constant stimuli task while the present study used an adaptive training task. Therefore, participants were trained at their individual threshold of performance. This might have contributed to the observed improvement across training sessions. The learning trajectory across training sessions was similar for the ITD and ILD groups in the present study. Klingel et al. (2021) also observed similar trajectories across training sessions (namely no change) for the two groups, but a stronger improvement from the pretest to the first training session that partly dissipated in the posttest in the ILD group while the ITD group showed a weaker improvement that remained constant through to the posttest. However, due to the differences between the training and the testing task in the present study (i.e., an adaptive training vs. a constant-stimuli testing task), it was not analyzed whether this pattern replicates.

### Binaural-cue weight measurement

The current study also introduced a new method for measuring binaural-cue weights. Traditionally, binaural-cue weights have been measured using ITD/ILD trading ratios by fixing one of the cues and letting the participant adjust the other cue until the auditory image is centered (e.g., Deatherage & Hirsh, 1959). However, this method leads to a stronger weighting of the to-be-adjusted cue, either because of an attention shift (Lang & Buchner, 2008) or cue-specific adaptation (Moore et al., 2020). Estimating binaural-cue weights based on the lateralization of stimuli with spatially inconsistent ITD and ILD (e.g., Macpherson & Middlebrooks, 2002) is not susceptible to this bias. This approach, however, requires sophisticated equipment to accurately record response locations, such as virtual reality equipment. Furthermore, Klingel et al. (2021) observed response compression (i.e., responses closer to the midline) from pre- to posttest using the lateralization method, potentially complicating the interpretation of results. This does not happen in the discrimination task since no lateralization responses are given. Also, the present method is not dependent on accurate virtual space simulation, as the lateralization method might be. And, similar to the lateralization training and other “open loop” methods (Stecker, 2010), it is neither susceptible to an attentional bias as no cue is actively manipulated nor to cue-specific adaptation as both cues change from trial to trial. Instead, it only requires a simple left/right response and, therefore, does not need sophisticated equipment and instead can be run on a regular desktop computer, tablet, or cell phone.

### Limitations and future directions

For lateral sources close to the head, ILDs do not only indicate the source’s azimuth but also change according to the distance of the sound source with larger ILDs indicating sources closer to the head (Shinn-Cunningham et al., 2000). For lateral azimuths on the right, increasing the ILD may, therefore, either be perceived as movement to the right (assuming equal distance of the two stimuli) or movement towards the ear, which would be to the left along the interaural axis. This ambiguity may have increased the noise in the responses for lateral azimuths, but it should not systematically affect the pre- vs. posttest comparison in binaural-cue weights, especially since we observed the post–pre P_ILD_ difference to be largely azimuth independent.

We presented auditory stimuli without HRTF filtering via headphones. This was done to prevent access to monaural spectral localization cues, which might also provide information about the stimulus azimuth and in turn prevent purely binaural-cue reweighting. Kumpik et al. (2010), for example, found stronger weighting of unaltered monaural compared with binaural cues instead of a change in the binaural-cue weighting after modifying the binaural cues while preserving monaural cues at one ear. However, as monaural and binaural localization cues interact in everyday life, the effect of binaural-cue reweighting in more realistic conditions and for different stimuli is an interesting topic for future studies. For example, while Klingel and Laback (2022) established the need for specific frequencies to induce binaural-cue reweighting, only noise stimuli were used. Testing other stimuli that do not transmit fine-structure cues and thus should be usable for reweighting experiments, such as amplitude modulated or vocoded stimuli, might inform us about potential applications. It would also be interesting to clarify under which conditions binaural-cue reweighting and binaural-to-monaural-cue reweighting occurs for azimuthal sound localization. Additionally, it is unclear whether the lack of externalization resulting from the exclusion of spectral cues affected the binaural-cue weighting. Kumpik et al. (2019) used HRTFs as well as reverberation to promote externalization and found stronger ILD weighting for their broadband stimuli compared with Macpherson and Middlebrooks’ (2002) wideband stimuli that included HRTFs but no reverberation. Therefore, the higher ILD weights in Kumpik et al. (2019) likely resulted from the added reverberation, which makes ITDs less reliable (Rakerd & Hartmann, 2010), rather than from HRTFs or externalization. Nevertheless, future studies are needed to systematically disentangle the effects of these sound properties on binaural-cue weighting.

While the ecological relevance of binaural-cue reweighting in the normal auditory system may be limited due to its dependence on the auditory stimuli (Klingel & Laback, 2022), namely, the lack of reweighting for stimuli including low-frequency temporal-fine-structure information that is often available in real-life sounds, the results may be relevant for hearing-impaired or cochlear-implant (CI) listeners. Listeners with sensorineural hearing loss, for example, may not have access to fine-structure ITD cues, while retaining some sensitivity to envelope ITD cues (Lacher-Fougère & Demany, 2005). CI listeners also seem to have access to envelope ITD cues only. Many CI stimulation strategies use high-rate constant pulse trains and encode ITDs only via the envelope of the stimulus waveform. Furthermore, even when ITDs are encoded via the pulse timing, CI listeners’ sensitivity pattern resembles the pattern for envelope ITDs in acoustic hearing (Bernstein & Trahiotis, 2002; Laback et al., 2007). In fact, binaural-cue reweighting has been observed in CI listeners using the lateralization task when ITDs were encoded via the pulse timing of low-rate pulse trains (Klingel & Laback, 2021).

Since the posttest was performed immediately after the final training session, the present data does not give any insight on how long the observed effects might persist while experiencing natural binaural cues, or how much stronger/long-lasting the effect might be if more training sessions were performed. Considering that Klingel et al. (2021) observed that part of the reweighting effect in the ILD group already got lost from the last training session to the posttest (but also note that this was not the case for the ITD group), it is likely that the effect does not persist over longer periods of time in which participants experience natural (i.e., consistent) binaural cues. With respect to the potential of binaural-cue reweighting for CI listeners, the goal should therefore be to use the training to get accustomed to future stimulation strategies encoding ITD cues more saliently, meaning that CI listeners would continue to receive reinforcement in their every-day life.

### Summary and conclusions

The present results suggest that binaural-cue reweighting can be induced with a simple left/right discrimination task, which might make a training more easily accessible for a wide range of listeners (e.g., after introducing a previously impeded cue to hearing devices such as cochlear implants, or even for normal hearing listeners who might not be using the optimal cue weighting in varying environments).

## Footnotes

1 Xie's estimation was based on the head-related transfer functions (HRTFs) of the KEMAR head model with DB-61 small pinnae, considering a source distance of 1.4 m. Also note that the ITD thresholds for fine-structure ITDs (i.e., ITDs conveyed by the temporal fine structure of the signal) become unmeasurable above approximately 1.4 kHz (Brughera et al., 2013) where the envelope ITDs (conveyed by the amplitude modulations in the signal envelope) dominate. However, Bernstein and Trahiotis (1982) showed that low-frequency residual energy far below the nominal pass band of a stimulus can provide salient ITD cues, even if those cues are transmitted at a low sensation level. Nevertheless, the fact that we observe reweighting suggests that participants were using envelope-ITD cues (Klingel & Laback, 2022) even though we cannot rule out residual contribution of fine-structure cues.
